# Composite Hydrogels in Three-Dimensional *in vitro* Models

**DOI:** 10.3389/fbioe.2020.00611

**Published:** 2020-06-16

**Authors:** Zhitong Zhao, Catarina Vizetto-Duarte, Zi Kuang Moay, Magdiel Inggrid Setyawati, Moumita Rakshit, Mustafa Hussain Kathawala, Kee Woei Ng

**Affiliations:** ^1^School of Materials Science and Engineering, Nanyang Technological University, Singapore, Singapore; ^2^Environmental Chemistry & Materials Centre, Nanyang Environment and Water Research Institute (NEWRI), Nanyang Technological University, Singapore, Singapore; ^3^Skin Research Institute of Singapore, Singapore, Singapore; ^4^Center for Nanotechnology and Nanotoxicology, Harvard T.H. Chan School of Public Health, Harvard University, Boston, MA, United States

**Keywords:** 3D *in vitro* model, composite hydrogel, extracellular matrix mimicking, bioprinting tissue-like constructs, regenerative medicine

## Abstract

3-dimensional (3D) *in vitro* models were developed in order to mimic the complexity of real organ/tissue in a dish. They offer new possibilities to model biological processes in more physiologically relevant ways which can be applied to a myriad of applications including drug development, toxicity screening and regenerative medicine. Hydrogels are the most relevant tissue-like matrices to support the development of 3D *in vitro* models since they are in many ways akin to the native extracellular matrix (ECM). For the purpose of further improving matrix relevance or to impart specific functionalities, composite hydrogels have attracted increasing attention. These could incorporate drugs to control cell fates, additional ECM elements to improve mechanical properties, biomolecules to improve biological activities or any combinations of the above. In this Review, recent developments in using composite hydrogels laden with cells as biomimetic tissue- or organ-like constructs, and as matrices for multi-cell type organoid cultures are highlighted. The latest composite hydrogel systems that contain nanomaterials, biological factors, and combinations of biopolymers (e.g., proteins and polysaccharide), such as Interpenetrating Networks (IPNs) and Soft Network Composites (SNCs) are also presented. While promising, challenges remain. These will be discussed in light of future perspectives toward encompassing diverse composite hydrogel platforms for an improved organ environment *in vitro*.

## Introduction

*In vivo*, cells are embedded within a complex 3D microenvironment composed of combinations of extracellular matrix (ECM) components, biological factors, neighboring cells etc. (Benders et al., 2013; Huang et al., 2017). Under this microenvironment, cells are constantly spreading, migrating, proliferating, differentiating, and interacting with each other and their surroundings in response to biological stimuli. 2D (monolayer) cell cultures as the common practice in cell-based assays are simple, high throughput options for various biomedical research purposes (Ashammakhi et al., 2018). However, researchers have been aware of the limitations of 2D compared to 3D cultures since the 1970s (Elsdale and Bard, 1972). 2D cell culture assays may provide misleading and non-predictive data as they are unable to capture the anatomical and biochemical complexities of native tissues and organs (Horrobin, 2003; Hogenesch and Nikitin, 2012; Edmondson et al., 2014). Therefore, animal tests are usually conducted after 2D cell culture studies, before clinical trials. Unfortunately, animal models are time consuming, expensive, raises ethical dilemmas and are often limited by species-specific anatomy and physiology (Elliott and Yuan, 2011). For decades, 3D *in vitro* models have captured the imagination of scientists since they could mimic some of the structural and functional characteristics of native tissues and organs (Sart et al., 2014; Knight and Przyborski, 2015; Bersini et al., 2016). Their 3D microenvironment enable cells to interact with neighboring cells and matrix components in all directions (instead of directly interacting with a synthetic hard plastic surface in the case of 2D cultures), and in doing so, guide cellular behavior and functions under more physiologically relevant conditions (Alhaque et al., 2018; Kaushik et al., 2018; Hong et al., 2019). Thus, 3D *in vitro* models are viable alternatives to animal studies to screen biochemical compounds for drug development. They also offer the opportunity to understand the biological processes of cells, tissues, and organs *in vitro*, and have been applied in the fields of tumor research, tissue generation, disease screenings, and more (Huh et al., 2011; Ranga et al., 2014; Sambale et al., 2015; Alhaque et al., 2018; Drost and Clevers, 2018). So far, numerous 3D *in vitro* models have been developed, including organoids (Yin et al., 2016; Drost and Clevers, 2018), cellular spheroids (Baraniak and Mcdevitt, 2012; Laschke et al., 2013; Nguyen et al., 2018) cell-laden biomimetic constructs (Ng and Hutmacher, 2006; Kang et al., 2016; Vo et al., 2016) and organs-on-chips (Huh et al., 2011; Polini et al., 2014).

The essence of developing 3D *in vitro* models is to build tissue- or organ-like constructs that have similar structural and/or functional characteristics as real tissues or organs with the recapitulation of multiple cell type interactions and biological responses. Thus, a matrix that resembles most closely the features of native ECM, either from the onset or over the course of a culture period, is key. To replicate Nature, what better way is there than to look into Nature itself for solutions? One does not need to look far to realize that the blueprint used repeatedly by Nature to produce the optimal ECM to support tissue and organ development is that of composite hydrogels. The soft, viscoelastic dermis made from proteoglycans-filled interpenetrating networks of collagen, elastin, and fibronectin, and the hard and tough cortical bone made from highly crosslinked organic fractions of collagen, proteoglycans, and glycoproteins reinforced with inorganic hydroxyapatite deposits are but a couple of examples. From a materials design point of view, native ECMs of living tissues are immaculately orchestrated composite hydrogels in which fibrous networks, typically collagen, are embedded into soft hydrated polysaccharides and glycosylated protein matrices, with biological macromolecules interspersed within (Burla et al., 2019; Freedman and Mooney, 2019). Besides providing the necessary biochemical cues, the consequent mechanical properties customized to the functional requirements of the tissues, are ascribed to this composite structure (Sharma et al., 2016). Not surprisingly, hydrogels have been used extensively *in vitro* as ECM-like matrices to mimic the biological environment that cells experience within native tissues (Oliva et al., 2017). They can hold large amounts of water or biological fluids without losing their structure due to their 3D, hydrophilic, crosslinked polymeric networks, which resemble the hydrated nature of native ECM. Hydrogels fabricated from synthetic polymers could possess similar and reproducible mechanical properties as that of native tissues (Sahiner, 2013; Yu et al., 2019), while hydrogels fabricated from natural biopolymers, especially proteins, can present bioactive ECM components to cells (Mohammed and Murphy, 2009; Antman-Passig and Shefi, 2016; Kim S. H. et al., 2018). Hydrogels can be designed and fabricated *via* chemical (e.g., free radical polymerization, various addition reactions and Redox reactions) and physical (e.g., ionic interactions, hydrogen bonding, and crystallization) crosslinking methods (Hennink and van Nostrum, 2002; Jin et al., 2013; Lowe, 2014). Importantly, hydrogels crosslinked under mild conditions would allow for the encapsulation of cells with high cell viability during the fabrication of biomimetic constructs (Yang et al., 2017). Therefore, hydrogels fabricated from purely synthetic or natural polymers are hardly able to meet all structural and functional requirements as a biomimetic tissue-like 3D construct. Synthetic hydrogels, such as polyethylene glycol (PEG) (Cha et al., 2011), poly(vinyl alcohol) (PVA) (Tominaga et al., 2008), and polyacrylamide (PAm) (Han et al., 2017a,b) have the versatility to be tuned in terms of physical and chemical properties *via* varying molecular weights or crosslinking degrees. However, they lack the ECM components such as cell adhesion motifs that can modulate cell behaviors and functions. Hydrogels fabricated from cell-friendly, nature-derived biopolymers such as gelatin and collagen are also limited in use due to their poor mechanical properties and batch-to-batch discrepancy (Helary et al., 2010; Zhao et al., 2016; Annabi et al., 2017; Liu et al., 2018; Freedman and Mooney, 2019). Therefore, composite hydrogels with more than one constituent have been developed in order to overcome the drawbacks of each component.

Generally, there are several strategies of producing composite hydrogels such as physical blending (Liu et al., 2013), *in situ* synthesis (Wang et al., 2009; Huang et al., 2012), bio-conjugation (Ahadian et al., 2015), and forming Interpenetrating Networks (IPN) (Chen et al., 2015; Nonoyama et al., 2016; Fares et al., 2018; Karami et al., 2018) and others. For example, Liu et al. blended titania nanosheets into aqueous solution of a water-soluble vinyl monomer (i.e., N-isopropylacrylamide), and then the hydrogelation was induced by light (λ > 260 nm) (Liu et al., 2013). Huang et al. fabricated a hydrogel matrix such as poly(2-hydroxyethyl methacrylate) (pHEMA), then forced calcium and phosphate ions to diffuse into the hydrogel 3D networks to induce biomimetic mineralization of calcium phosphate *in situ*. With this method, the authors formed various nanoarchitectures including nanoscale fibers, sheets, and needles by adjusting the molecular chemisty, through changing functional groups, on pHEMA (Huang et al., 2012). Bioconjugation is a stretegy for introducing cell-responsive components into hydrogel networks to improve their biological performance. With this approach, proteins or peptides such as growth factors and cell adhesion motifs could be grafted onto polymer backbones *via* covalent bonds (e.g., dislufide exchange, ester formation, click chemistry) or non-covalent bonds (e.g., ionic interaction) to form the composite hydrogels for regulating cell behaviors (Hoffman, 2012; Ahadian et al., 2015; Martino et al., 2015). Fares and co-workers formed IPN composite hydrogels comprising of a pectin grafted polycaprolactone crosslinked by calcium ions and a gelatin methacryloyl (GelMA) crosslinked by photo-induced radical polymeration. The results showed that this IPN hydrogel was cytocompatible and could support the growth of preosteoblasts *in vitro* (Fares et al., 2018). Additionally, extensive studies have also been performed to develop composite hydrogels that combine various natural biopolymers such as proteins (e.g., collagen, gelatine, keratin, and silk fibroin) (Ding et al., 2013; Su et al., 2016; Fares et al., 2018) and polysaccharides (e.g., hyaluronic acid, chitosan, and alginate) (Min et al., 2015; Si et al., 2017; Qu et al., 2019) with synthetic polymers. Based on a more traditional composite technology approach, filler materials in the form of engineered nanomaterials (ENMs) have been introduced to fabricate composite hydrogels with improved mechanical properties to match that of native ECMs in terms of stiffness, viscoelasticity etc. (Thoniyot et al., 2015; Mehrali et al., 2017; Aviv et al., 2018; Gan et al., 2020). All these types of composite hydrogels have great potential as biomimetic matrices in 3D *in vitro* models and will be discussed further subsequently.

Fabrication techniques should be considered hand-in-hand with the composite strategy. In the human body, tissues and organs have highly ordered macro- and micro-architectures that support the complex interplay between cells, ECM, and a milieu of biomolecules within the environment (Huang et al., 2017). From developmental biology, we know that tissue morphogenesis happens bottom up over an extended period of time and is intricately coordinated between the different components in a highly dynamic process. This same process cannot be replicated outside the body with current technologies. Traditional approaches to fabricate 3D matrices such as porogen leaching, freeze drying, and gas foaming are limited in possibilities for the simultaneous incorporation of cells (Hamasaki et al., 2008; Bai et al., 2015; Bhardwaj et al., 2015; Scotti and Dunand, 2018). Technologies to build *in vitro* models at the microscale, such as biomedical microelectromechanical systems (bio-MEMs), cannot recreate the dynamic, multicellular, architectures of tissues, and organs (Pati et al., 2016). In recent times, bio-printing methods have been reinvented to create physiologically relevant 3D models through simultaneous or sequential arrangement of cells, materials, and biological factors mixed in a crosslinkable bio-ink. Bio-printing technologies have been used extensively to pattern cells and bioactive agents within hydrogels to fabricate 3D *in vitro* models (Levato et al., 2014; Pedde et al., 2017; Kim et al., 2019). However, achieving microscale resolution is a challenge.

It is clear that 3D is better than 2D in replicating more physiologically relevant models *in vitro*. It is also becoming apparent that composite hydrogels are growing in significance in our efforts to perfect this replication. In this Review, we therefore specifically aimed to present the most relevant and recent studies describing composite hydrogel designs and their adoption for constructing 3D *in vitro* models. We focused on summarizing the various types of composite hydrogels, highlighting their reported applications, and pointing out their limitations. Clearly, challenges remain. We point these out as well and provide our thoughts on future trends and developments in this exciting space.

## Types of 3D *in vitro* Models

So far, numerous 3D *in vitro* models have been developed, such as cellular spheroids (Ho et al., 2016; Laschke and Menger, 2017; Bin et al., 2018; Li and Kumacheva, 2018), organoids (Fatehullah et al., 2016; Bartfeld and Clevers, 2017; Cruz-Acuña et al., 2017; Kratochvil et al., 2019), cell-laden biomimetic constructs (Levato et al., 2014; Koo et al., 2018), mini-organs (Kang et al., 2016; Noor et al., 2019), organs-on-a-chip (Inamdar and Borenstein, 2011; Bhatia and Ingber, 2014; Polini et al., 2014; Takebe et al., 2017) etc. Among them, organoids are multicellular systems formed through cells differentiation and self-organization of pluripotent stem cells or tissue-derived progenitor cells to model the features of tissues or organs in an *in vitro* setting (Hu et al., 2018), while cellular spheroids are mainly used to describe 3D aggregates of cells (Ong et al., 2018).

**Figure 1:**
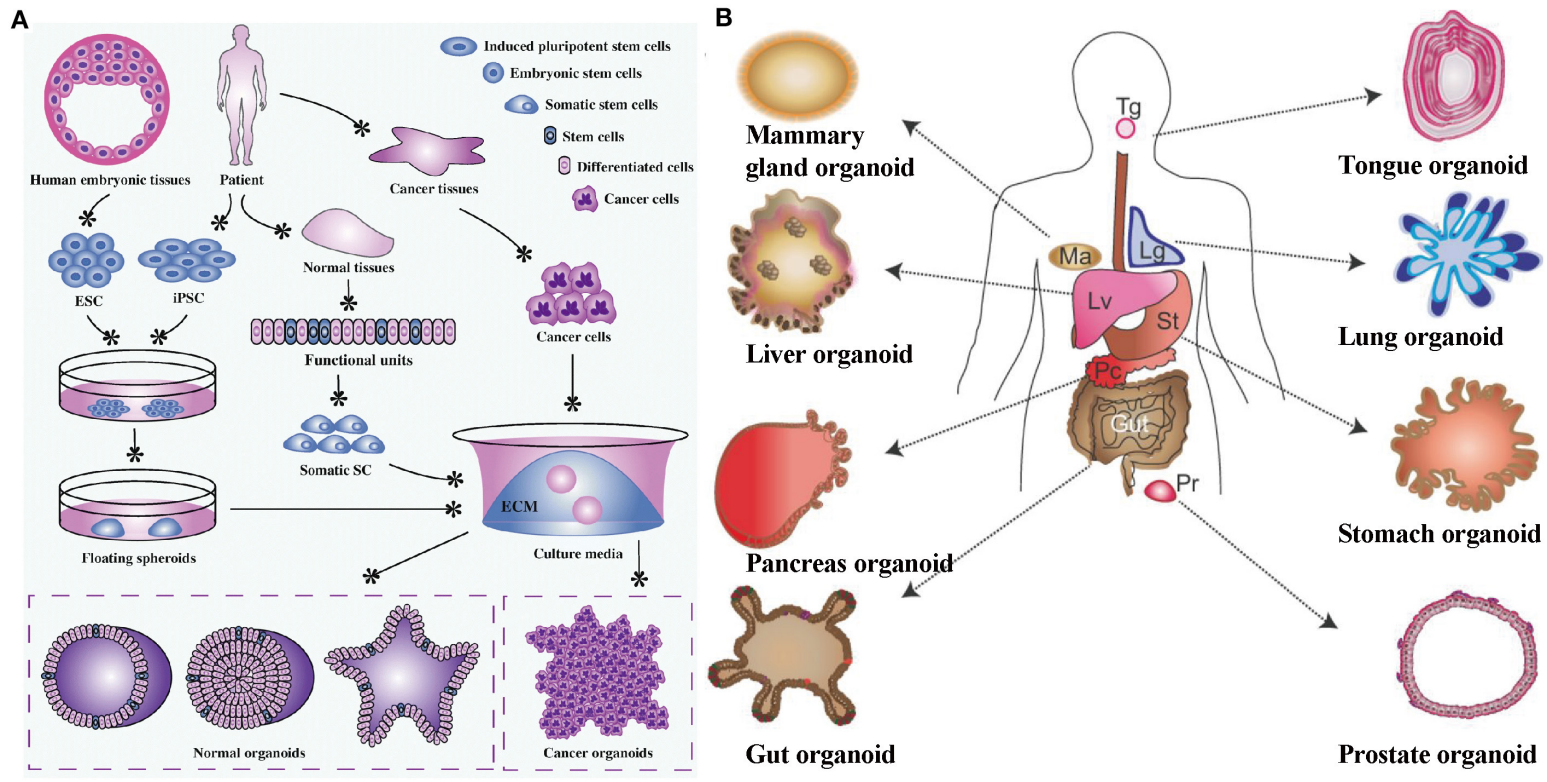
**(A)** Organoid establishment from stem cells and cancer cells. Embryonic stem cells from human embryonic tissues and induced pluripotent stem cells from adult tissues firstly experience directed differentiation, generate floating spheroids, and subsequently are planted on extracellular matrix in specific culture medium to initiate organoid culture. Primary tissues from patients can be dissociated into functional units, which contain somatic stem cells. These somatic stem cells are enriched and cultured in three-dimensional medium to form organoids. Tumor cells isolated from cancer tissues can also form tumoroids in well-defined three-dimensional culture. Reproduced under the terms of the CC BY Creative Commons Attribution 4.0 International License (Xu et al., 2018). **(B)** Various types of organoids derived from both isolated adult stem/progenitor cells or from isolated fragments of tissue from the corresponding organ (e.g., intestinal crypts, liver or pancreas ducts). Lg, Lung; Lv, Liver; Ma, mammary gland; Pc, Pancreas; Pr, Prostate; St, Stomach; Tg, Tongue. Reproduced with permission from COMPANY OF BIOLOGISTS (Huch and Koo, 2015).

Spheroids and organoids are 3D microphysiological systems that are formed *via* one or more cell types proliferating, differentiating, and self-organizing within close proximity to one another, forming complex and organized cell structures that recapture some structural and functional features of real organs (Clevers, 2016). This strategy started as a method to produce cancer cell spheroids for studying tumorigenesis and cancer drugs (Leong and Ng, 2014). In recent times, this strategy has been used more regularly for 3D tissue and organ reconstruction. As shown in Figure 1, functional tissue spheroids and organoids can be formed using various stem cell sources with the capacity to differentiate into nearly any tissue type (Drost and Clevers, 2018; Hu et al., 2018; Xu et al., 2018). Organoids cultured from pluripotent stem cells (PSCs) contain multiple cell types can be an advantage when trying to mimic the multicellular complexity of native tissues. In addition, organoids can also be derived from organ or tissue fragments. For example, intestinal organoids can be generated through the expansion of biopsies of intestinal tissue, which contains intestinal stem cells (Dekkers et al., 2013). Once the cell source is chosen, the subsequent protocols are mostly similar, in which a homogenous culture medium is used for culture. The cells are required to be suspended or encapsulated in a 3D environment that allows them to freely grow and remodel their environment, while engaging in self-directed cell sorting without any guidance from investigators (Yin et al., 2016). Free growth can be accomplished by culturing cells in low attachment conditions (e.g., low attachment microplate, hanging droplet) (Ng et al., 2007; Turner et al., 2016), so-called scaffold-free method, or in a naturally derived hydrogel (scaffold-base method) such as a decellularized ECM. Under low attachment conditions, stem cells are forced to suspend in the medium in the form of clusters, before differentiating and proliferating to form organoids. In the scaffold-base method, the naturally derived ECM are often used as a supporting matrix, which is capable of instigating necessary instructive signaling. ECM components, such as laminin and fibronectin, possess integrin receptors of cells to maintain cell identity and functions during formation of organoids. For instance, the Engelbreth-Holm-Swarm (EHS) matrix, a gelatinous protein mixture harvested from mouse sarcoma cells, has been widely used for organoid cultures (Jo et al., 2016; Sampaziotis et al., 2017; Lee et al., 2018). This matrix is well-known by the trade name Matrigel®. However, animal-derived matrices suffer from batch-to-batch variability and cannot be easily tailored to obtain unique organoid niches for specific organs (Kleinman and Martin, 2005). Hence, recent efforts have shifted to engineering biomaterials that can support and promote organoid formation, because they can provide a chemically-defined matrix that allows for precise tuning of matrix properties to guide cell fates (Gjorevski et al., 2016; Cruz-Acuña et al., 2017; Kratochvil et al., 2019). According to the Review by Kratochvil et al., the properties of engineered biomaterials could have impacts on organoids formation such as the presence of cell-adhesive ligands, mechanical properties (e.g., stiffness, stress relaxation, stress stiffening), matrix geometry, degradation etc. (Kratochvil et al., 2019). Although organoids lack complete structural and functional features of real tissues or organs, their relative ease of bio-assembly, reproducibility, and the ability to capture cellular heterogeneity allows them to be suitable platforms for screening drugs and diseases as alternative to 2D cell-based assays and animal models (Clevers, 2016; Nguyen et al., 2018). A variety of organoid types and combinations have been explored in the literature, including but not limited to skin (Lee et al., 2018), intestine (Ootani et al., 2009), liver (Zhang R.R. et al., 2018), kidney (Takasato et al., 2015; Taguchi and Nishinakamura, 2017), lung (Dye et al., 2015), pancreas (Dorrell et al., 2014), and various types of brain tissues (Pasca et al., 2015; Qian et al., 2016).

Organs-on-chips are microfluidic devices for growing cells in continuously perfused, micrometer-sized chambers in order to model physiological functions of tissues or organs. This technology is to build minimal units that recapitulate some functions of real tissues or organs (Polini et al., 2014). The simplest organ-on-a-chip system is a single, perfused microfluidic chamber containing one type of cells that reproduce specific functions of one tissue (Polini et al., 2014). In a complex design, two or more micro-chambers are connected by porous membranes (Bhatia and Ingber, 2014). They are lined on opposite sides containing different types of cells to recreate the interfaces between tissues. These systems can also involve physical forces, such as fluid shear stress at a physiologically relevant level, cyclic strain, and mechanical compression (Booth and Kim, 2012). The introduced physical forces can permit analysis of organ-specific responses, including recruitment of circulating immune cells, in reaction to the environmental perturbations (George et al., 2018). Organs-on-chips are particularly well-suited to the study of biological phenomena that depends on tissue microarchitecture and perfusion. Organs-on-chips technology offers the great opportunity to investigate the basic mechanisms of organ physiology and disease for screening drugs and understanding metastasis of cancer cells (Polini et al., 2014; Skardal et al., 2016). Researchers have fabricated chips for the study of the liver (Prodanov et al., 2016), kidney (Maschmeyer et al., 2015), intestine (Kim et al., 2012), lung (Huh et al., 2007), heart (Vollert et al., 2014), skin (Sriram et al., 2018) etc. over the past decade. These existing models are successful in recreating specific individual aspects of organ function, but are too simplified when compared to the entire organ physiology. Thus, various types of organoids could be incorporated into organ-on-a-chip systems to create more complex and physiologically relevant tissues or organs in the future (Maschmeyer et al., 2015).

**Figure 2:**
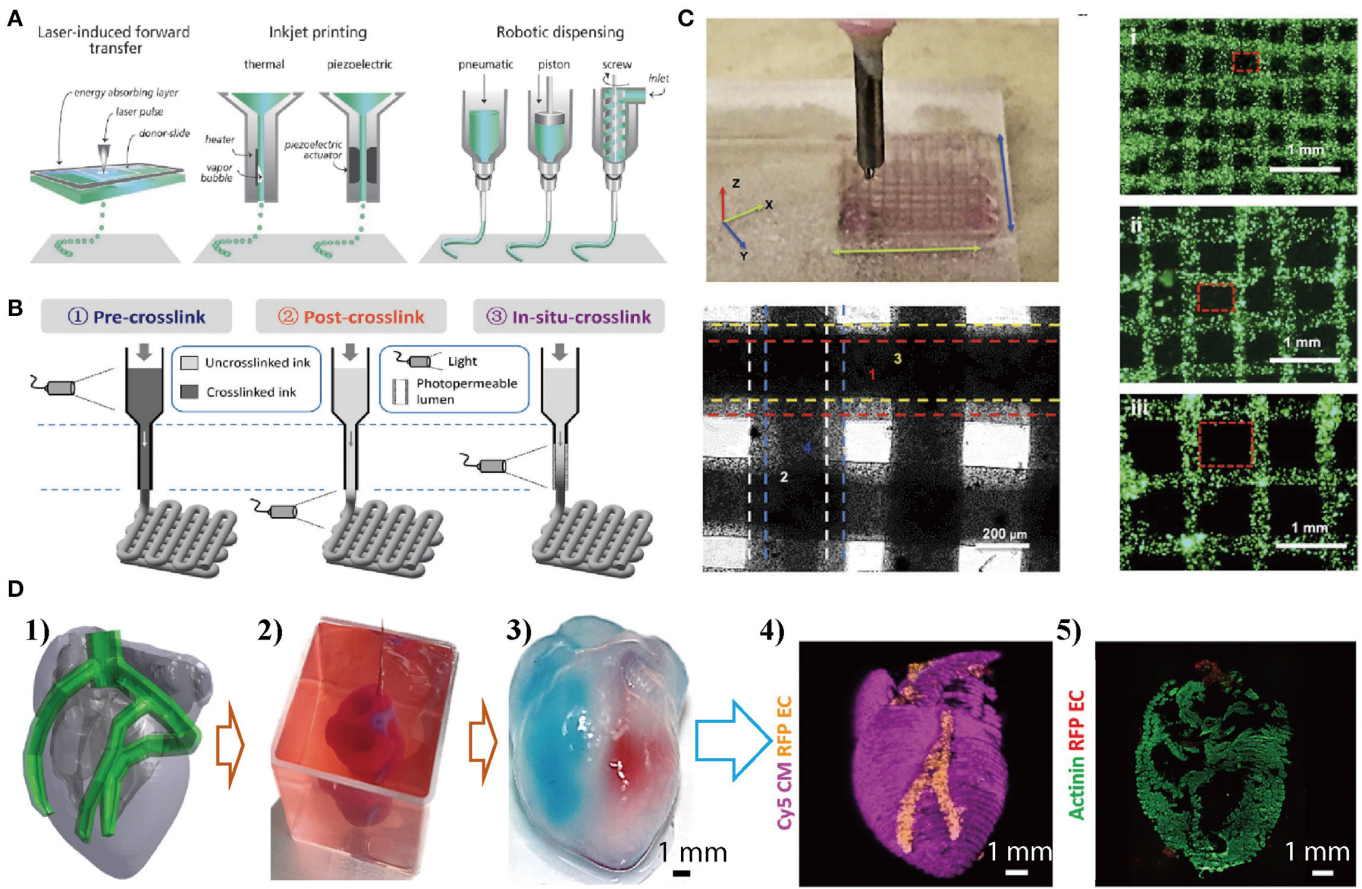
Bio-printing cell-material composites layer-by-layer to produce mini-organs with complex structures. **(A)** Cell-laden composite hydrogels are created by applying laser light (laser-induced forward transfer) or by extrusion (inkjet printing with or without robotic dispensing). Reproduced with the permission from WILEY-VCH (Malda et al., 2013). **(B)** Light-induced crosslinking strategies for the bioprinting of photocrosslinkable bioinks. Reproduced with permission from WILEY-VCH (Ouyang et al., 2017). **(C)** Bio-printed simple 3D constructs using gold/GelMA composite bioinks, with different grid architecture. Reproduced with the permission from WILEY-VCH (Zhu et al., 2017). **(D)** Bio-printed 3D heart models with two types of cells: (1) The human heart CAD model. (2) A printed heart within a support bath. (3) After extraction, the left and right ventricles were injected with red and blue dyes, respectively, demarcating the hollow chambers and presence of the septum. (4) 3D confocal laser microscopy image of the printed heart (cardiomyocytes in pink, endothelial cells in orange). (5) Cross-section of the heart immunostained for sarcomeric actinin (green). Reproduced under the CC BY Creative Commons Attribution 4.0 International License (Noor et al., 2019).

Cell-laden hydrogel constructs and the tissue- and organ-like miniatures have been extensively investigated in the field of tissue engineering and have been by far the most successful method to produce functional tissues for clinical transplantation (Bin et al., 2018; Park et al., 2018; Feng et al., 2019; Williams, 2019). Cell-laden tissue- or organ-like constructs are formed by suspending cells in a gel matrix to mimic the structural and functional characteristics of real tissues and organs. The gel matrices should be cell-friendly, capable of incorporating bio-macromolecules to guide cell fates, and suitable to be processed with cells to create similar architectures to that of real tissues and organs. For this purpose, a plethora of materials have been used, including natural occurring and synthetic ones (Caliari and Burdick, 2016). However, the real tissues and organs have more complex architectures and components (e.g., different types of cells, proteins, and signaling molecules) than the tissue-like constructs. This complexity poses a problem in fabrication due to the lack of appropriate technologies to simultaneously control the spatial arrangement of gels, cells, and other biological components. Now, 3D-bioprinting technologies may be a solution as it could create complex tissue-like constructs through precise placement of cell-laden hydrogels in a layer-by-layer fashion (Malda et al., 2013; An et al., 2015; Zhai et al., 2018; Zhang Y. S. et al., 2018; Zhang et al., 2019). Cell-laden hydrogel constructs can be bioprinted by applying laser light or by extrusion (inkjet printing) as illustrated in Figure 2. In bio-printing, the synthetic or natural materials used to recreate tissues-like constructs together with cells and biomolecules are termed as bio-inks (Gungor-ozkerim et al., 2018). The suitability of hydrogels as bio-inks is dependent on their physicochemical properties, e.g., rheological properties and crosslinking mechanisms (Foyt et al., 2018). Crosslinking of hydrogels can be triggered before (pre-crosslink), after (post-crosslink), or during (*in situ* crosslink) extrusion shown in Figure 2 (Moroni et al., 2018). Among the crosslinking methods, light-triggered radical polymerisation has mostly been used and evolved into a very powerful and flexible tool to fix the structures or shape of the bio-printed constructs (Zhao et al., 2016). Novel bio-inks that make use of self- and co-assembly of combinations of materials to produce composite hydrogels are also emerging (Hedegaard et al., 2018). Current hydrogels developed as bio-inks to create tissue-like constructs include gelatin, collagen, alginate, hyaluronic acid (HA), and PEG-derivate polymers etc. (Jang et al., 2018). During bio-printing, the vitality of encapsulated cells should also be prioritized as many factors could result in low cell viability such as the shear force and harmful crosslinking methods (e.g., UV light). Bio-printing methods have successfully fabricated cell-laden constructs from the ones with simple layer-by-layer structures to mini organs having complex structures (e.g., heart shown in Figure 2). Although several types of tissue- or organ-like constructs were successfully fabricated *via* the bio-printing method, they are relatively simple, having only one or two cell types, in one or two matrix materials (Ashammakhi et al., 2019). Nonetheless, while bio-printing functional tissues and organs for clinical transplantation has not become a reality, the method has provided new impetus for the evolvement of more sophisticated *in vitro* models that bring us closer to real tissues and organs in a dish.

## Composite Hydrogels

As mentioned in the Introduction, hydrogels are, in many ways, akin to the native ECM, resulting in their preference as ECM-like matrices for cellular support as well as their functions in 3D *in vitro* models. However, single component hydrogels are not able to capture all functions and properties of native ECM. Thus, composite hydrogels containing two or more constituent materials with synergistic properties have been developed to better support cells in 3D models. In this section, composite hydrogels are categorized and discussed based on their constitutive materials.

### Nanomaterial Incorporated Composite Hydrogels

**Figure 3:**
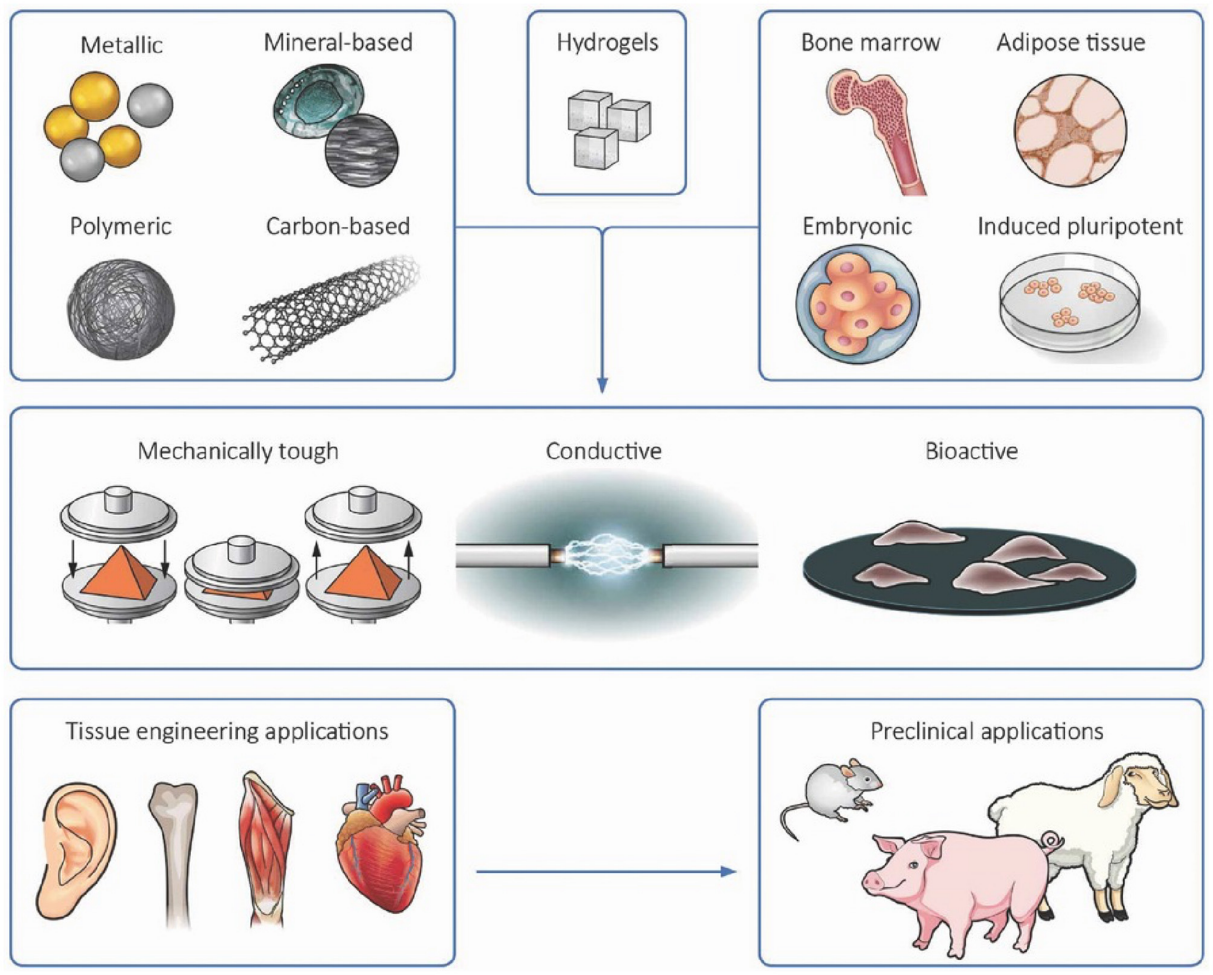
Incorporation of nanomaterials into cell-laden hydrogels presents a versatile strategy for engineering 3D tissue-like constructs with enhanced functionalities including increased bioactivity, electrical conductivity, and mechanical strength. Reproduced with permission from WILEY-VCH (Mehrali et al., 2017).

Nanomaterials incorporated into hydrogels come in the forms of nanoparticles (NPs), nanofibers, and nanotubes (Sharifi et al., 2012; Chimene et al., 2015). Nanomaterials have been commonly incorporated into hydrogels to create unique and potentially useful properties that are not found in the original hydrogel. Indeed, the majority of nanomaterials incorporated into hydrogel matrices have improved their mechanical properties; some of them also exhibited electrical and biological activities (Allo et al., 2012; Mehrali et al., 2017). Incorporation of nanomaterials into cell-laden hydrogels to reinforce their physiochemical and biological properties also presents a versatile strategy for engineering multifunctional constructs for tissue engineering (as illustrated in Figure 3) (Mehrali et al., 2017). In this section, several types of nanomaterials including metals, metal oxide NPs, carbon-based nanomaterials, polymer nanofillers etc. are presented as enhancing components in hydrogels. While promising, the safety and long-term risks of using nanomaterial incorporated composite hydrogels in the body will need to be more carefully studied. This is especially so in cases where the nanomaterials are expected to persist in the body.

#### Metal and Metal Oxide NPs

As reviewed, silver (Ag) NPs have been extensively studied as antibacterial agents and incorporated into various synthetic hydrogels such as PAm, polyacrylic acid (PAA), and PVA to form composite hydrogels to prevent bacterial contamination during biomedical applications (Thoniyot et al., 2015). The methods used to combine Ag NPs with hydrogels, including simple mixing, *in situ* synthesis, and chemical crosslinking etc. (Thoniyot et al., 2015). *In situ* synthesis methods could circumvent the issue of NPs aggregation resulting from simple mixing methods. For example, small Ag NPs (2–5 nm) with uniform distribution have been obtained within hydrogel matrix *via* this method by researchers (Murthy et al., 2008). Importantly, the *in situ* synthesis method is versatile and can also be allied to many other types of NPs synthesis within hydrogel matrix such as iron (II, III) oxide (Fe_3_O_4_) (Helminger et al., 2014), Gold (Au) (Sahiner, 2013), and ZnO (Zhao X. et al., 2018). Apart from synthetic hydrogel, the strategy can also be applied to naturally derived polymers such as carbohydrate, chitosan, and gelatin to obtain biocompatible, degradable NPs/hydrogel composites. These composite hydrogels could be applied in biomedical fields such as an implantable dressing for skin wound healing (Thoniyot et al., 2015). Au NPs exhibit the antibacterial properties as well but is rarely used due to high cost. They can also cause localized heating in hydrogels *via* irradiation of light at their plasmonic peak, a phenomenon that may be exploited for remote-controlled drug release from a temperature-responsive hydrogel matrix (Li and Mooney, 2016). Other metal NPs such as nickel (Ni) can be incorporated into hydrogel matrices for the development of magnetic field-driven actuators. For example, Fuhrer et al. incorporated Ni NPs by crosslinking them into pHEMA hydrogel backbone *via* covalent bonding of the particles, which prevented the leaching of metals out of the hydrogel. The Ni NPs/pHEMA composite hydrogel afforded very strong magnetic actuators with high mechanical stability, elasticity, and shape memory effect (Fuhrer et al., 2009).

Metal oxide NPs such as zinc oxide (ZnO), iron oxide (γ-Fe_2_O_3_/Fe_3_O_4_), silica (SiO_2_), and others, have also been combined with hydrogels to fabricate composite hydrogels to enhance their antibacterial, ferromagnetic, and semi-conducting properties. For example, Xiang et al. designed an injectable hydrogel through rapid assembly of dopamine and folic acid crosslinked by transition metal ions and introduced ZnO NPs into the hydrogel. They found that reactive oxygen species generated within the hydrogel matrix made it a great antibacterial composite against *Staphylococcus aureus* (Xiang et al., 2019). Schwartz et al. incorporated ZnO NPs into PNIPAAm hydrogels, which exhibited excellent bactericidal behavior toward *Escherichia coli* with a concentration of ZnO as low as 0.74 mmol/ml (Schwartz et al., 2012). Yadollahi et al. synthesized ZnO NPs *in situ* within chitosan hydrogel matrices during the formation of hydrogel. They found that the composite had higher swelling ratio than the pure hydrogel in different aqueous solutions, and had potential for drug delivery (Yadollahi et al., 2016). Apart from NPs, metal oxide can also be made into nanofibers before combining with hydrogels. For example, Si et al. introduced electro-spun SiO_2_ nanofibers to create a nanofibrous composite hydrogels by crosslinking them with alginate. The nanofibrous composite hydrogels exhibited ultrahigh water content (99.8 wt%), complete recovery from 80% strain, and injectability (Si et al., 2017). Iron oxide (Fe_3_O_4_ and γ-Fe_2_O_3_), a commonly synthesized ferromagnetic material, has recently been incorporated into hydrogels to form ferrogels as actuators to mimic human muscle movement (Li et al., 2013). Skaat et al. fabricated a series of magnetic fibrin hydrogel scaffolds by incorporating fibroblast growth factor (FGF)-conjugated γ-Fe_2_O_3_ NPs. They demonstrated that the FGF-conjugated NPs significantly improved the growth of nasal olfactory mucosa cells seeded in the magnetic scaffold (Skaat et al., 2012). Xu et al. incorporated magnetic NPs into a PEGDA and GelMA hydrogel to form micro-scale hydrogel composites. This material retained the biocompatibility of the hydrogels, while contributing additional capabilities for magnetic manipulation. These microscale gels can be used as building blocks to create 3D complex layer-by-layer constructs with encapsulating cells under external magnetic fields. By spatially controlling the magnetic field, the authors could manipulate 3D construct geometries and achieve multilayer assembly of microgels (Xu et al., 2011).

#### Carbon-Based Nanomaterials

Carbon-based nanomaterials including carbon nanotubes (CNTs), graphene, graphene oxide (GO) etc. have been widely applied in engineering and medical fields (Xin et al., 2018). Carbon-based materials can act as near-infrared (NIR) light absorbing or electrically conductive materials in the hydrogel matrix (Serrano et al., 2014). For example, Arslantunali et al. fabricated CNTs/hydrogel composites to regulate nerve cells activity. The neuro-blastoma cells exhibited healthy state with wide extension and ideal communication on the composite (Arslantunali et al., 2014). Zhao et al. combined CNTs with chitosan to design an injectable, antibacterial conductive composite hydrogel, which showed robust mechanical strength, rapid shape recovery, and high blood uptake capacity. The chitosan functionalized with glycidyl methacrylate formed hydrogels *via* radical polymerization. The developed CNTs/chitosan had better blood-clotting ability, higher blood cell and platelet adhesion than gelatin sponge and gauze. They also had a better healing performance than commercial Tegaderm™ films when used for skin wound healing (Zhao Z. et al., 2018). Graphene has shown unique advantages in significantly improving the combinatorial properties of traditional hydrogels (Liao et al., 2018). For example, Tai et al. obtained GO/PAA composite hydrogels *via in situ* free radical polymerization method at low temperature. The composite hydrogel displayed excellent swelling characteristics and electrical response as compared to the pure PAA (Tai et al., 2013). Liu et al. fabricated GO/PAm composite hydrogels *via* the similar *in situ* polymerization method, showing that the tensile strength of GO/PAm hydrogel was about 4.5 times higher than the pure PAm with a 30-time breaking elongation (Liu et al., 2012). Liang et al. designed a series of GO/HA composite hydrogels for skin wound dressing. They prepared the hydrogels based on dopamine grafted-HA and reduced GO using a H_2_O_2_/horseradish peroxidase system. These hydrogels exhibited high swelling, degradability, tuneable rheological property with similar mechanical properties to human skin. This type of wound dressing also exhibited antioxidant activity, tissue adhesiveness, haemostatic ability, and self-healing ability due to the presence of polydopamine (Liang et al., 2019).

#### Polymeric Nano-Fillers

This type of nanomaterials includes micelles, dendrimers, hyper-branched polymers, polymer nanofibers, and nanocrystals. For example, Zhong et al. incorporated polyamidoamine dendritic NPs into collagen hydrogel matrices to enhance their biological stability (Zhong and Yung, 2009). Zhang et al. achieved a week-long, controlled release of active ingredients by blending hyper-branched polyamine ester NPs within a hydrogel network (Zhang et al., 2013). Qu et al. designed an injectable hydrogel containing polymer micelles for repairing skin damage. The copolymer micelle was cross-linked to chitosan, which showed improved mechanical properties similar to that of skin in terms of modulus and tensile strength, adhesiveness and self-healing ability. The hydrogels also exhibited efficient haemostatic performance and biocompatibility during wound healing (Qu et al., 2018). Li et al. incorporated polycaprolactone (PCL) electrospun nanofibers into HA hydrogels by forming interfacial covalent bonds between maleimide-PCL fiber fragments and thiol-HA crosslinked network *via* click chemistry. This composite hydrogel had a very high bulk porosity and storage modulus that could match the mechanical properties of adipose tissue. Importantly, this fibrous hydrogel allowed infiltration of host macrophages and differentiated them into the pro-regenerative phenotype after subcutaneous injection in a rat model. These polarized macrophages secreted pro-angiogenic cytokines and growth factors to promote the formation of vascularized tissue (Li et al., 2019). Polylactic acid (PLA) nanofibers were also incorporated into alginate hydrogel by Narayanan et al. to prepare composite hydrogels containing human adipose-derived stem cells. The results showed that cell proliferation within the hydrogel matrix could be promoted by the nanofibers (Narayanan et al., 2016). Nanocellulose, as a class of natural sustainable materials derived from plants or bacteria, has been widely used in the fields of biomedical, energy, construction etc. (Ding et al., 2018; Kontturi et al., 2018). Incorporation of cellulose nanofibers (CNF) within polymer matrices could create tough and flexible hydrogels for specific biomedical applications. Many studies have shown that CNFs are compatible with a wide variety of synthetic and natural polymers such as PVA, PAm, chitosan, collagen, alginate, and gelatin to improve their physicochemical properties as well as their performance across a wide range of applications as reviewed by Nascimento et al. (2018). In addition, another form of nanocellulose [nanocrystals (CNC)] was found to confer significant shear-thinning property to the bio-ink as viscosity decreased significantly when shear rate was increased. The shear-thinning property enabled the use of CNC/hydrogel bio-inks for printing 3D cell-laden constructs (Markstedt et al., 2015).

Other than the aforementioned nanomaterials, quantum dots (Chang et al., 2009), nano-calcium phosphate (e.g., hydroxyapatite) (Kim et al., 2014), nano-clay (Han et al., 2017a), and others have also been explored to improve the physical and biological properties of hydrogels for biomedical applications and have been reviewed elsewhere (Mehrali et al., 2017; Timofejeva et al., 2017; Kuśtrowski, 2018).

### Composite Hydrogels With Biological Factors

In the native ECM, there are many biomacromolecules including growth factors, cell adhesion motifs, enzymes, and other signaling molecules, which collectively control cell fates in a precisely controlled manner (Huang et al., 2017). However, synthetic polymers such as PEG, PVA, and PAm, as well as nature derived materials such as alginate and chitosan, do not possess the biological cues of the mammalian ECM. Significant efforts have gone into combining relevant ECM components into synthetic and natural hydrogels to render them suitable as ECM-like matrices to culture cells (Cambria et al., 2015; Murphy and Lampe, 2015). In this regard, tethering proteins or peptides with integrin-binding sequences to the polymer backbone by forming covalent or non-covalent bonds is a common strategy (Dalby et al., 2014). The cell adhesion peptide sequences used include fibronectin-derived RGD and LDV, and laminin-derived IKVAV and YIGSR (Santiago et al., 2006). Interestingly, Lee et al. could pattern RGD sites into a collagenase-sensitive hydrogel to direct cell migration *via* a photon laser scanning photolithography technique. They proved that tissue regeneration could be guided at the microscale level within 3D scaffolds by spatially providing appropriate bioactive cues (Lee et al., 2008). Growth factors can be introduced into hydrogels to stimulate cell growth, proliferation, and differentiation, especially for wound healing. Vascular endothelial growth factor (VEGF) has been shown to be one of the key players in the wound healing process by promoting angiogenesis and is often introduced into engineered transplants for tissue engineering (Freudenberg et al., 2015). Byambaa et al. chemically conjugated VEGF onto an injectable gelatin hydrogel to treat bone non-union defects. The VEGF was grafted onto -COOH modified gelatin by forming ester bonds *via N*′-ethylcarbodiimide hydrochloride (EDC)/N-hydroxysuccinimide (NHS) coupling. The growth and differentiation of encapsulated cells (osteogenic and endothelial cells) were significantly enhanced by this strategy (Byambaa et al., 2017). In another example, Lee et al. bio-printed neural stem cells-containing collagen hydrogels 1–2 mm apart from VEGF-containing fibrin gels into an artificial neural tissue. Results showed that cells preferentially migrated toward the fibrin gel presumably due to the presence of VEGF (Lee et al., 2010). In the study by Poldervaart et al., endothelial progenitor cell-laden Matrigel^®^ was bio-printed with two different regions: one region containing VEGF, and the other without the growth factor as control. The results demonstrated that cell migration and vascularization were significantly improved within VEGF regions in comparison with the control region (Poldervaart et al., 2014). Certain stem cells require extensive biochemical stimuli inherent to their natural ECM niche for proper differentiation. These biochemical stimuli may arise from cell adhesive molecules such as laminin and fibronectin or signaling ligands such as Transforming Growth Factor (TGF) and Bone Morphogenetic Protein (BMP) (Dalby et al., 2014; Freedman and Mooney, 2019). For example, Gurkan et al. incorporated TGF-β1 and BMP-2 into GelMA-based bioinks together with human mesenchymal stem cells (MSCs). They printed this bio-ink into constructs with a gradient of growth factors to mimic the fibro-cartilage transition at the bone-tendon interface, leading to differentiation of human MSCs toward the osteogenic and chondrogenic phenotypes in a spatially defined manner (Gurkan et al., 2014). *In vivo*, bioavailability of growth factors is tightly regulated by non-specific associations between the factors and ECM glycosaminoglycans, through affinity binding domains. Thus, Martino et al. modified fibrin hydrogels with an oligopeptide binding domain derived from fibronectin to conjugate growth factors such as VEGF and BMP. The results showed that this modified hydrogel required far less additional growth factor supplementation than unmodified hydrogels when recruiting and guiding stem cell differentiation (Martino et al., 2011).

### Composite Hydrogels Comprising Natural and Synthetic Polymers

Due to inherent limitations, hydrogels fabricated from natural biomaterials or synthetic polymers lack the ability to accurately mimic all the features of native ECM. Other natural polymers such as collagen and gelatin have batch-to-batch discrepancies, poor mechanical properties and poor biological stability (Pradhan et al., 2016). Composite hydrogels comprising of natural and synthetic materials could combine the best of both worlds: the advantages of natural polymers (such as cell-adhesive ligands and filamentous structure) and synthetic polymers (good mechanical properties and tuneable chemical properties) (Afewerki et al., 2019). There are huge numbers of reports describing the functionalities and advantages of such blends, referred to as double networks or interpenetrating networks (IPNs) containing synthetic-synthetic, synthetic-natural, and natural-natural hydrogels (Chen et al., 2015; Nonoyama et al., 2016; Zhang et al., 2016). In this section, only composite hydrogels combining natural-natural polymers and natural-synthetic polymers that were used as matrices to support cell growth and functions or as bio-inks to fabricate cell-laden 3D constructs *via* bio-printing, are reviewed.

Alginate is a seaweed-derived and anionic polysaccharide, which is very suitable for bio-printing owing to its high biocompatibility and rapid crosslinking ability (Hartrianti et al., 2017; Zhang and Khademhosseini, 2017). Alginate hydrogels are normally crosslinked by exposure of alginate to CaCl_2_ solution at ambient temperature with sodium-calcium ion exchange reaction occurring (Chan and Mooney, 2013). Although alginate has similarities with glycosaminoglycans in the ECM, it lacks bioactivity, thereby it is often combined with nature-derived proteins for biomedical applications. For example, Chaudhuri et al. combined alginate with Matrigel^®^ and investigated how hydrogel stiffness affects the malignant progression of normal mammary epithelium, by controlling the degree of ionic crosslinking of alginate with Ca^2+^ ions. It was found that an increase in matrix stiffness induced the malignant cell phenotype in mammary epithelium (Chaudhuri et al., 2016). Shin et al. incorporated marine collagen and agarose with alginate to fabricate a physically crosslinked, bioactive hydrogel for 3D cell cultures. This composite hydrogel exhibited excellent cytocompatibility for various cell types. Multicellular spheroids cultured within this composite hydrogel resulted in high yields (Shin et al., 2016). Additionally, gelatin having RGD sequence can also be combined with polysaccharide to improve their bioactivity (Afewerki et al., 2019). Chung et al. bio-printed a construct using alginate/gelatin mixed solution as bio-inks. This printed construct exhibited similar mechanical properties to those of pre-crosslinked alginate but were more superior in supporting cell growth (Chung et al., 2013). Chitosan is another attractive polysaccharide due to its biocompatibility, biodegradability, and antimicrobial ability (Mohanty et al., 2018). However, chitosan suffers from slow gelation and poor bioactivity, which could be solved with the addition of gelatin. Because chitosan is positively charged, it can interact with negatively charged gelatin at pH 6.5 to form polyelectrolyte complexes. Ng et al. found that this polyelectrolyte hydrogel could provide high shape fidelity for the printed 3D constructs and good biocompatibility with skin fibroblasts, suggesting its suitability for bio-printing with cells (Ng et al., 2016). In addition, gelatin can also be mixed with other natural proteins such as silk fibroin to form composite hydrogel. For the gelatin/silk fibroin composite, silk fibroin could provide superior mechanical properties and tuneable degradability, while gelatin having RGD sequences to improve cell adhesion. This composite hydrogel has been used as bio-inks together with chondrocytes for bio-printing constructs to repair damaged cartilage (Das et al., 2015). Bartnikowski et al. blended GelMA with gellan gum to tune the mechanical properties of hydrogels. The results suggest that this composite hydrogel could achieve the viscoelasticity of native cartilage by adjusting the proportion of gellan gum to GelMA (Bartnikowski et al., 2015). Feng et al. fabricated a composite hydrogel composing of thiolated gelatin and vinyl sulfonated HA *via* click chemistry between -SH and C=C bonds. They found that encapsulated bone MSCs in these hydrogels showed high viability, proliferation, and chondrogenic differentiation potential *in vitro* (Feng et al., 2019). Other forms of composite bio-inks are also being developed, such as a novel blend of synthetic amphiphilic peptides and naturally occurring keratin protein (Hedegaard et al., 2018). By making use of the self- and co-assembly potential of these materials, the study introduces a novel 3D-bioprinting platform capable of encapsulating and distributing cells within tuneable pericellular environments. PEG is a synthetic polymer and available in many chemical variants (linear or multi-arm) with different molecular weights. PEG variants such as PEGDA or methacrylate (PEGMA) have been widely used in fabrication of various medical products. PEG polymers have been combined with natural biopolymers such as gelatin, alginate and keratin to improve mechanical properties, biological activity as well as the printability of the hydrogels (Yue et al., 2018; Ashammakhi et al., 2019).

Inspired by the composite structure of soft tissues, where it is typical to find fibrous networks embedded in a weak hydrogel, the recently coined term of soft network composites (SNCs) emerged. SNCs are hydrogel-based composites specifically designed with a highly organized fibrous network made from synthetic polymers, reinforcing a soft hydrogel matrix. Early on, SNCs were fabricated as low-modulus thin films with mechanical responses that match the non-linear properties of human skin, for demonstration as skin-mounted electrophysiological sensors, as Jang et al. (2015) showed. Subsequently, varying strategies of using fibrous components such as PCL fibers have also emerged, in combination with PEG, HA, or alginate hydrogel matrices to fabricate composites for the improvement of mechanical properties (Shin et al., 2015; Li et al., 2019). However, these studies only used the fibers fragment produced by electrospinning as reinforcement filler and lacked the interconnectivity of a fibrous network within the hydrogel to elevate mechanical reinforcement. To obtain the continuous networks, Bas et al. introduced Melt Electrospinning Writing (MEW) by combining additive manufacturing and electrospinning principles. The authors first manufactured PCL fibrous networks using MEW, and then infused the hydrogel solution (PEG/heparin and fibrin hydrogel) into the networks before inducing gelation. They demonstrated that the composite hydrogels made with this approach could contain PCL fibrous networks with different pore size (fiber spacing), exhibiting mechanical anisotropy, viscoelasticity, and morphology analogous to those of native ECMs. Consequently, this fibrous network-reinforced composite hydrogel was demonstrated to provide suitable microenvironment for human chondrocyte culture and neocartilage formation *in vitro* (Bas et al., 2017). Visser et al. also used MEW to reinforce soft hydrogels of GelMA and alginate by introducing an organized, high-porosity PCL microfibre network. The results showed that the stiffness of the hydrogel-microfibre composites increased significantly by up to 54-fold in comparison with the bare hydrogel or microfibre network on their own (Visser et al., 2015). Burla et al. embedded collagen fibers into hyaluronan matrix and investigated their mechanics by combining mechanical measurements and computer simulations. They demonstrated that this composite hydrogel exhibited synergistic mechanical behavior with enhanced stiffness and delayed strain-stiffening (Burla et al., 2019). Overall, SNCs made from hydrogels reinforced with fibrous networks are promising but still at an early stage of development. Future studies targeting the correlation of fibrous network architecture and reinforcement mechanism will help guide matrix designs and customization. The remodeling of SNCs over time, especially when biodegradable polymer networks are used, will be important considerations for longer term applications. In addition, fundamental mechanisms of fiber-hydrogel binding, *via* the possible range of covalent and non-covalent interactions, should be better understood and exploited to further enhance the potential of this approach to produce composite hydrogel systems.

## 3D Models Cultured Using Composite Hydrogels

Herein, we focus on representative demonstrations in the past decade to construct 3D models of specific tissues/organs with the involvement of composite hydrogels as: (1) Bio-inks to print cell-laden tissue/organ-like constructs to support cells and their functions; (2) Functionalized matrices to guide cell and tissue morphogenesis, behavior, and organization into organoids.

### Skin

Skin equivalents were the first type of 3D *in vitro* skin model, which are normally constructed from layers of keratinocytes and fibroblasts to form the epidermis and dermis (Ng and Hutmacher, 2006; Sun et al., 2014). Typically, a collagen-based gel substrate containing dermal fibroblasts was first deposited, followed by a layer containing melanocytes and keratinocytes to mimic the architecture of native skin (Min et al., 2018). Collagen type I-based hydrogels are the most used substrate when producing skin equivalents (Kim B. S. et al., 2018). A wide range of products and strategies are now available for purposes of skin repair and grafting, toxicology testing and drug development (Shevchenko et al., 2010; Kathawala et al., 2019). Composite hydrogels are growing in popularity for skin regeneration because the native skin matrix is an orchestrated blend of several ECM components including collagen, elastin, fibronectin, vitronectin, glycosaminoglycans, and more. In a recent report, Hakimi et al. mixed fibrinogen, collagen, and sodium hyaluronate as a dermal layer that can be applied using a handheld bioprinter, and a layer containing fibrinogen and sodium hyaluronate as the epidermis. For printing sheets, a layer of thrombin was co-delivered to induce gelation of fibrinogen to stabilize the layer, before the slower thermally induced gelation of collagen occurs. They demonstrated that these biomaterial sheets could form *in situ* in murine and porcine excisional wound models, illustrating the capacity of deposition onto compliant wound surfaces of irregular topographies (Hakimi et al., 2018). A key limiting factor in current skin equivalent models is the lack of vasculature and functionally important appendages such as sebaceous glands (Gurtner et al., 2008; Kim B. S. et al., 2018). Skin organoid models derived from pluripotent stem cells cultured in Matrigel^®^ have been shown to produce sebaceous glands, adipocytes, and hair follicles in mice, over a 30 days *in vivo* period (Lee et al., 2018). However, Matrigel^®^ poses limitations of batch-to-batch discrepancy and high cost (Kleinman and Martin, 2005). Defined formulations of composite hydrogels such as those described above may provide a more sustainable delivery vehicle for skin organoids.

### Bone

Bone is a natural biocomposite that plays a vital role in supporting the body, protecting organs, and blood production (Zimmermann and Ritchie, 2015). To do these, bone has a unique hierarchical structural organization at a multi-scale (from nano to macro) that gives it the high strength and fracture toughness required (Reznikov et al., 2018). Using hydrogels to support the recreation of such a load bearing structure is a tall order. Nonetheless, 3D bio-printing using composite hydrogels consisting of alginate, gelatin, and hydroxyapatite to support mesenchymal stem cells (MSCs) for this purpose has been demonstrated (Wüst et al., 2014). Zhai et al. fabricated an osteoblast-laden nanocomposite hydrogel construct based on PEGDA, nanoclay, and HA. The construct showed excellent osteogenic ability in the long term, rationalized to be due to the optimal microenvironment induced by bioactive ions of nano-clay (Zhai et al., 2018). Besides single cells, MSC spheroids have also been investigated and combined with composite hydrogels for bone tissue engineering. For example, Ho et al. fabricated an RGD-modified alginate hydrogel and used it to culture MSC spheroids. They evaluated the effects of cell adhesion capacity in relation to bone-forming potential within the matrix. Results showed that MSC spheroids in the hydrogel presented higher cell survival rates and mineralization capacity than the unmodified alginate (Ho et al., 2016). Levato et al. also developed a novel composite hydrogel combining MSC aggregates-laden PLA microcarriers with GelMA/gellan gum as bio-inks to fabricate living bone constructs *via* bio-printing. This strategy allowed for extensive expansion of cells to achieve high cell concentrations within the matrix, and high cell viability. *In vitro* results also demonstrated that the strategy promoted MSC adhesion, osteogenic differentiation, and bone matrix deposition (Levato et al., 2014). Biological factor incorporated composite hydrogels have also been extensively explore for bone regeneration. In a more recent study, Maisani et al. developed a BMP-2 loaded, injectable glycosyl-nucleosyl-fluorinated based hydrogel that provides controlled release of the BMP-2 for optimal bone tissue regeneration. They demonstrated that this system could promote significant bone defect regeneration in calvarial bone defects in mice after 8 weeks of implantation, in comparison with direct application of BMP-2 solution without any hydrogel (Maisani et al., 2018).

### Cartilage

Cartilage is a relatively simple tissue anatomically, but regeneration is difficult due to the lack of vascularization. Regardless, due to the clinical demands from osteoarthritis, cartilage regeneration has attracted much focus (Benders et al., 2013; Levato et al., 2017). Numerous scaffold-based techniques that offer chondrocytes effective and realistic 3D microenvironments have been developed for this purpose over the years (Hutmacher et al., 2003). In particular, 3D cell-laden constructs are a facile strategy to treat cartilage defects when compared to cell-only transplantation. Not surprisingly, 3D bio-printing has become a popular approach for this purpose, in the last decade. Kang et al. prepared various composite hydrogels comprising of gelatin, fibrinogen, HA, and glycerol as bio-inks, and successfully fabricated a stable, human-scale ear-shaped cartilage *via* bio-printing. To facilitate the diffusion of nutrients, they embedded microchannels into the cell-laden tissue constructs. *In vitro* results demonstrated production of cartilaginous matrix in the constructs by 5 weeks. The constructs were also implanted into the dorsal subcutaneous space of mice and were found to support enhanced cartilage formation over 1 month (Kang et al., 2016). Shie et al. used a composite HA/polyurethane hydrogel to print 3D cell-laden constructs with MSCs for cartilage regeneration. The MSCs were found to differentiate successfully into chondrocytes, while the matrices were found to develop similar mechanical properties as that of articular cartilage (Shie et al., 2017). In yet another similar study, composite hydrogels comprising of PCL, alginate, and TGF were used to fabricate cartilage-like matrices *via* bio-printing with encapsulated chondrocytes. This composite construct with improved mechanical properties was found to enhance cartilage-like ECM deposition *in vitro* (Kundu, 2015).

### Liver

The liver is a key organ to detoxify chemicals and metabolize drugs in the body (performed mainly by hepatic parenchymal cells). 3D *in vitro* liver models could serve as a platform to investigate the physiological phenomena in the liver for the accurate predication of drug effects and toxic responses (Dorrell et al., 2014; Maschmeyer et al., 2015). Although the liver is capable of self-regeneration, it remains in high demand as an organ for transplantation in cases of severe damage (Dorrell et al., 2014). As such, there is a pressing need for the development of realistic 3D liver models. From a materials perspective, the lower mechanical demands of the liver make it an intriguing candidate for composite hydrogels to be applied. In a study by Ma et al., a mechanically compliant composite hydrogel was made from GelMA, as supporting matrices to induced PSCs (iPSCs)-derived hepatic cells, and glycidal methacrylate-HA, to promote endothelial cell proliferation. Using this composite formulation as the bio-ink, the team successfully bioprinted a patient-specific hepatic model that mimics the architecture and cell composition of native tissue (Ma et al., 2016). Mazzocchi et al. also prepared a blended hydrogel of HA and collagen to fabricate a liver-like construct *via* bio-printing, in which the native microenvironment properties were preserved. This liver construct contained primary human hepatocytes and liver stellate cells, and was demonstrated to be responsive to the effects of acetaminophen, a common liver toxicant (Mazzocchi et al., 2019). While Matrigel^®^ remains the typical choice for 3D liver cultures, most notably for liver organoids (Broutier et al., 2016), it is anticipated that composite hydrogels will rapidly emerge as common choices in the near future.

### Heart

The heart is mostly made up of cardiac muscles which are essentially intercalated cardiomyocytes embedded in an ECM of hyaluronan, fibronectin, fibrillin, proteoglycans, and collagens. Various composite hydrogels have been explored as matrices to support cardiomyocytes to fabricate heart tissue models. Gaetani et al. reported the fabrication of 3D bioprinted HA and gelatin composite patches containing progenitor cardiomyocytes. These were implanted into mice hearts with results showing good cell survival with increased cardiac and vascular differentiation markers after 4 weeks (Gaetani et al., 2015). Izadifar et al. incorporated carboxylated multi-walled CNTs into a collagen and alginate matrix to provide enhanced electrical, mechanical, and biological properties. With the concept of preparing a template to support pre-vascularized hybrid cardiac patches, the cardiac patches were cultured with human coronary artery endothelial cells which were found to exhibit good proliferation, migration, and differentiation within 10 days (Izadifar et al., 2018). Zhu et al. bioprinted a 3D cardiac tissue construct using Au nanorod-incorporated GelMA bioinks with cardiac cells encapsulated. Cell adhesion and organization were found to be improved by gold nanorods for cardiac tissue engineering (Zhu et al., 2017). Besides cardiac muscles, heart valves are also of interest clinically. Duan et al. employed bioprinting to fabricate a living heart valve with realistic anatomical architecture using an alginate/gelatin composite hydrogel. Aortic root sinus smooth muscle cells and aortic valve leaflet interstitial cells were successfully encapsulated into the construct. Results demonstrated that the tensile biomechanics of the cell-laden hydrogels were maintained, while acellular hydrogels exhibited reduced moduli and ultimate tensile strength (Duan et al., 2013). In another example of heart valve regeneration, Hockaday et al. used a PEGDA/alginate composite solution to print aortic valve scaffolds. It was showed that blended PEGDA/alginate hydrogels could achieve over 10-fold range in elastic modulus when compared with bare PEGDA. Porcine aortic valve interstitial cells were cultured within the constructs for 21 days (Hockaday et al., 2012). While these works demonstrate the potential of composite hydrogels for heart tissue regeneration and 3D cultures, successes are limited to demonstrations as relatively thin cardiac patches. The challenge will be to design more sophisticated composite matrices to support blood vessel ingrowth for better nutrients exchange, to produce thicker and larger functional constructs (Noor et al., 2019).

### Brain

The purpose of developing 3D brain cultures is primarily to understand the unique features of the human brain and to gain insights into neuropsychiatric disorders (Pas, 2018). 3D brain-like structures have been developed, which consist of individual layers of primary neural cells in hydrogels. For example, Lozano et al. printed a layered brain-like structure using an RGD-modified gellan gum as bio-ink, which encapsulates human cortical neurons. This RGD-modified hydrogel improved neuron cell proliferation and formation of neural networks, resulting in a complex, layered and viable 3D cell structure. This study offers the opportunity to reproduce more accurate 3D *in vitro* microstructures with applications ranging from cell behavior studies to improving the understanding of brain injuries and neurodegenerative diseases (Lozano et al., 2015). Brain organoids are often used for the modeling of neurological diseases *in vitro*, which can also be fabricated within the matrix of composite hydrogels. For example, Lindborg et al. designed a hyaluronate/chitosan hydrogel and seeded human PSCs within the matrix. After 10 days of culture, brain organoids spontaneously formed. The formed organoids had rosettes and neural-tube-like structures, and displayed physiological changes in intracellular Ca^2+^ concentration in response to the neurotransmitters glutamate and potassium (Lindborg, 2016).

### Intestines

**Figure 4:**
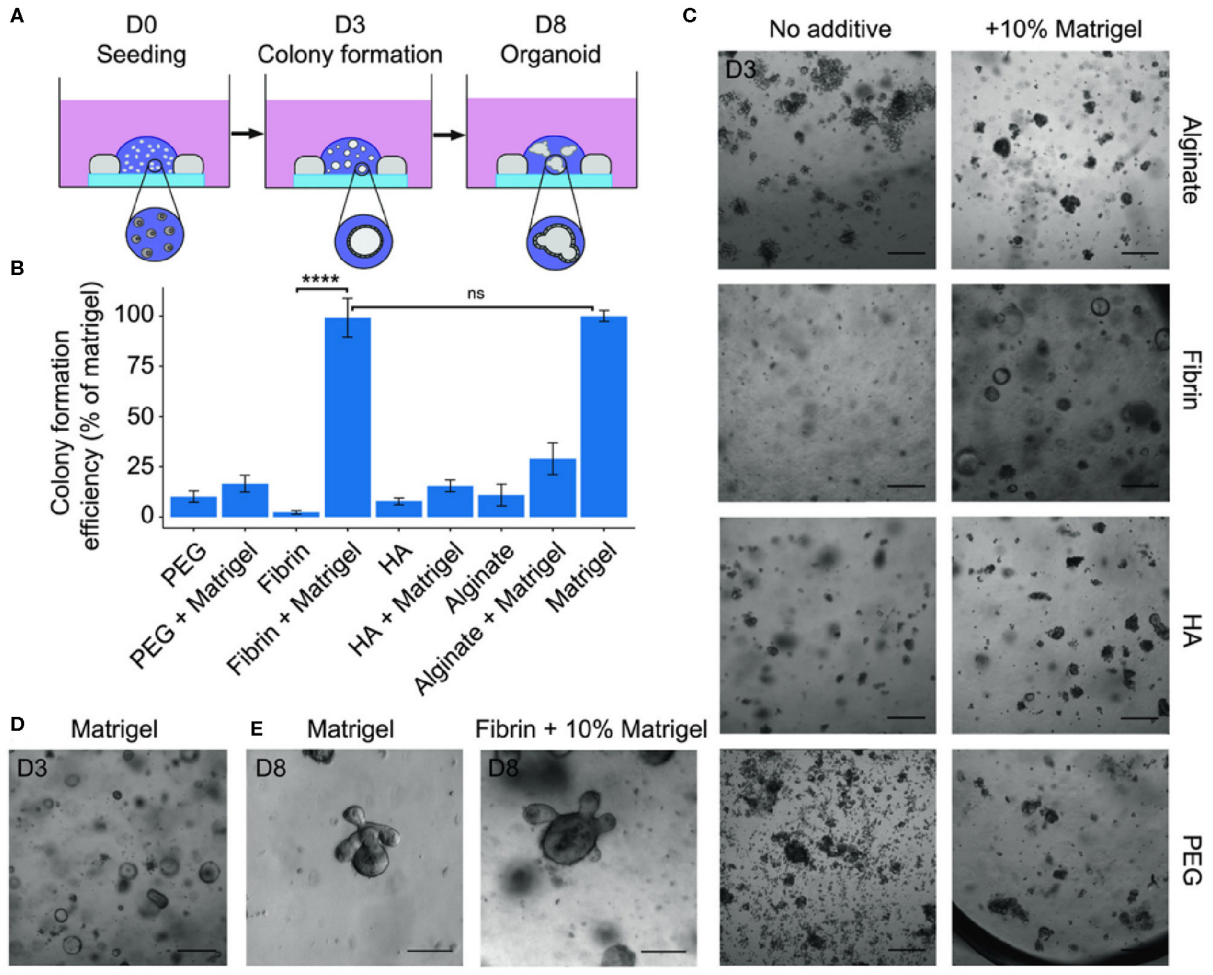
**(A)** Scheme of seeding assay showing the hydrogel inside a silicone mold on a glass cover slip. Small intestinal stem cells first grow as cystic spheres as seen at day 3 (D3) followed by the formation of budding organoids after ~1 week. **(B)** Quantifications of colony formation efficiency of mouse small intestinal stem cells in different hydrogels with or without 10% Matrigel. Data are expressed as a percentage of the colony count in Matrigel (100%). **(C,D)** Representative brightfield images of the cultures in the different hydrogels and Matrigel control. Scale bars: 200 μm. **(E)** Budding organoids as seen in Matrigel and in fibrin + 10% Matrigel after 8 days. Scale bars: 100 μm. Reproduced under the CC BY Creative Commons Attribution 4.0 International License (Broguiere et al., 2018).

The gastrointestinal tract (GIT) is a popular target of 3D cultures because of the significant interest and demands for food science and toxicology analyses, stem cell biology, and disease understanding. Within the GIT, 3D intestine cultures are the most developed for physiologic modeling of intestinal response to stimuli. Thus far, engineered biomaterials including composite hydrogels have been explored for intestinal organoid cultures as a replacement for Matrigel^®^-based matrices (Cruz-Acuña et al., 2017). Gjorevski et al. designed a PEG/laminin composite hydrogel containing the RGD cell adhesion peptide. The designed matrices were initially optimized for intestinal stem cell expansion and were subsequently found to be permissive to cell differentiation and intestinal organoid formation. With this study, the authors created a well-defined alternative to Matrigel^®^ for the culture of mouse and human stem cell-derived intestinal organoids (Gjorevski et al., 2016). Broguiere et al. developed a series of well-defined composite hydrogels to improve intestinal organoids formation and expansion (Figure 4) (Broguiere et al., 2018). They found that the fibrin/laminin matrix supported long-term expansion of all tested murine and human epithelial organoids, suggesting that this composite hydrogel could be an equivalent to Matrigel^®^. Work by Cruz- Acuña et al. similarly confirmed the suitability of cell adhesive ligand functionalized PEG hydrogel in supporting robust and highly reproducible human intestinal organoids (HIO) expansion. They also demonstrated that this hydrogel could serve as an injection vehicle that could be delivered into injured intestinal mucosa resulting in HIO engraftment and improved colonic wound repair (Cruz-Acuña et al., 2017).

### Cancer Models

Despite major advancements in therapy, cancer remains one of the leading causes of death in the world. Conventional 2D models of cancer cells and tissues cannot closely mimic the native tumor microenvironment (Benien and Swami, 2014). Therefore, there is a need to develop 3D cancer models with more physiologically relevant characteristics (Li and Kumacheva, 2018). *In vitro* cultures of cancer spheroids were in fact one of the first 3D cultures to have been successfully demonstrated (Weaver et al., 1997; Hutmacher, 2010). Not surprisingly, in recent years, bioprinted cancer models have been attempted and demonstrated to mimic the complexity of the native tumor tissues, and can be used for drug testing and to model cancer pathology *in vitro* (Shafiee and Atala, 2016). As with examples above, composite hydrogels have featured as bio-inks for this purpose. In a report by Zhao et al., a fibrinogen-gelatin-alginate composite bio-ink containing cervical cancer cells (HeLa) was printed to form a porous 3D architecture, in which the cancer cells were supplied with oxygen. Heat and CaCl_2_ solution were used to crosslink gelatin and alginate, respectively. The chemo-sensitivity of paclitaxel on HeLa cells within the 3D constructs was found to increase compared with 2D cultured cells (Zhao et al., 2014). Beck et al. introduced the composite hydrogel PEG/Matrigel^®^ as a platform to investigate cancer cell metastasis. In the study, rigidity of the matrix was tuned by varying the crosslinking density of PEG, while cell adhesive signals were incorporated into the PEG networks using peptide-conjugated cyclodextrin. In this model, it was found that the adhesive PEG networks induced dissemination of malignant mammary epithelial cells at intermediate values of adhesion and rigidity (Beck et al., 2013). Work done so far has demonstrated the versatility of using composite hydrogels for cancer studies. Given the complexities and variabilities of tumor microenvironments, future work could focus on patient-specific cancer cells which can provide more insights on the progress, diagnosis, and treatment of cancer diseases (Drost and Clevers, 2018; Li and Kumacheva, 2018). The interaction of cancer cells with new combinations of materials to replicate the cancer cell niche, through the use of novel composite hydrogels, is especially intriguing.

## Challenges and Future Perspectives

3D tissue and organ platforms that model complex physiological and pathological processes of native tissues and organs have enormous potential to be used for: (1) Safety and efficacy test of bioactive agents; (2) Understanding of pathogenesis and basic biology; (3) Clinical transplantation. These could immensely benefit our society by revolutionizing pharmaceutical, toxicological, and therapeutic applications. 3D *in vitro* models are not only more relevant alternatives to 2D cell cultures, they are also realistic replacements for animal models, which have come under significant criticisms in recent times due to ethical issues, high costs, and most important, irrelevance to human physiology in many scenarios. As presented above, numerous 3D *in vitro* models have been developed (Moroni et al., 2018), and composite hydrogels have been used extensively to support these models. Simply put, composite hydrogels are the logical choice to support 3D tissue and organ cultures because native tissues are inherently networks of various materials in a high water content environment.

**Figure 5:**
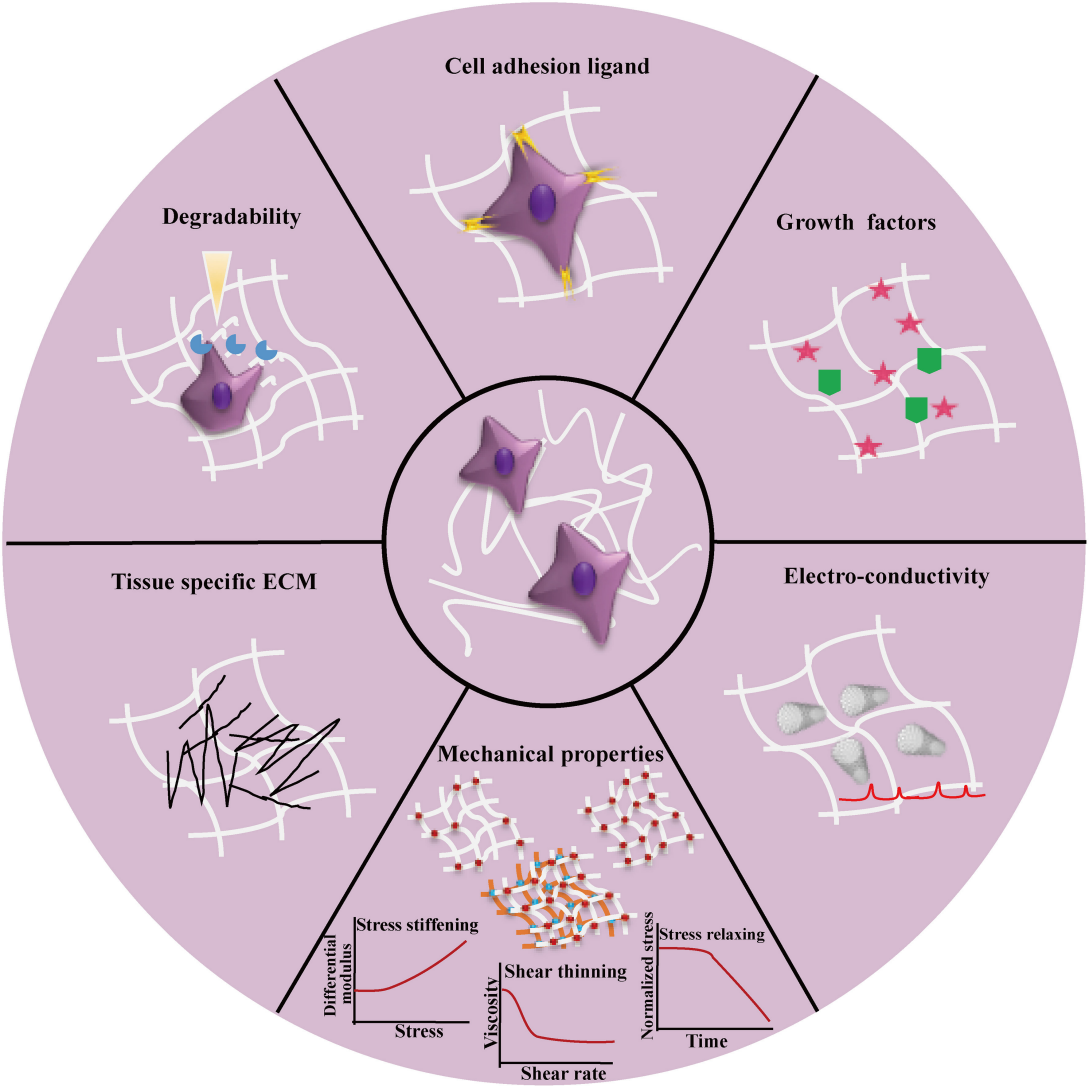
Overview of the design considerations for recreating 3D cell microenvironment *in vitro* by using composite hydrogels to fully mimic the features of native tissues. The design considerations can generally be divided into two tracks, i.e. biochemical (e.g., cell adhesion ligands, soluble factor immobilization and tissue specific ECM involvement) and biophysical design considerations (e.g., mechanical properties including tuneable strength requirements by crosslinking and involvement of multiple interpenetrating networks, stress-relaxing, stress-stiffening, degradability, and electrical conductivity).

Composite hydrogels are mainly used to recreate the natural cell microenvironment by mimicking the complexity of native ECM in an *in vitro* tissue model. ECM-mimicking matrices should not only support cell growth and functions but also provide the necessary biochemical and biophysical cues to regulate cell behaviors and fates in the same scenario as *in vivo*. Figure 5 shows the key design considerations of composite hydrogels to recreate 3D cell microenvironment of native tissues in 3D *in vitro* models. In this direction, biochemical cues present in ECM such as cell-adhesion ligands and growth factors could be introduced into the composite hydrogels to guide cell behaviors and fates. However, these biochemical cues are not uniformly distributed in native cell microenvironment. Thus, in future studies, they could be patterned within hydrogel matrices in a gradient over hydrogel backbones, for regulating cell behaviors (Lee et al., 2015). Native ECM can be degraded and remodeled by cell secreted proteases, giving rise to dynamic interactions between cells and the ECM (Oliva et al., 2017; Lueckgen et al., 2019). Similarly, dynamic cues such as proteolytic bonds could be incorporated into composite hydrogels to create a dynamic matrix for guiding cell and tissue development. In addition, reversible crosslinks could be incorporated into composite hydrogels to control mechanical deformations and degradation for supporting complex cell activities and long-term cell function (Wang and Heilshorn, 2015; Konieczynska and Grinstaf, 2017). The future composite hydrogels could also be designed to better mimic the mechanical properties of native ECM—viscoelastic and with stress relaxation and/or stress-stiffening properties, which are still limited in many current composite hydrogels (Deforest and Tirrell, 2015; Chaudhuri et al., 2016; Nam et al., 2019). In this respect, SNCs are an emerging trend that could escalate our ability to recapitulate the mechanical behavior of native ECMs. For an even more biomimetic and dynamic 3D *in vitro* model, smart materials with stimuli-responsive capabilities through chemical, electrical or mechanical signals will enable on-demand control to emulate microenvironment and ECM changes such as those seen during wound healing or tumorigenesis. As tissue and organ-like cultures become more and more realistic with the help of composite hydrogels, integrating different formulations of composite hydrogels could become necessary, for example, to incorporate blood vessels, nerves and lymphatic components into the cultures. From a materials processing perspective, the compatibility of composite hydrogel processing parameters, such as crosslinking mechanisms, may become a challenge. The complexity of crosslinking mechanisms could also depress the spatial and temporal resolution of bio-printing methods or reduce cell viability. While composite hydrogels can comprise of many types of materials, challenges in defining the balance between material concentrations, density of grafted functional groups/biomolecules will emerge, to achieve optimal results for remodeling native cell microenvironment and guiding cell behaviors.

Depending on the purpose of the 3D cultures and the complexity of native tissues, it may not be necessary, or impossible based on current technology, to replicate the exact native cell microenvironment. A tiered approach could be developed to identify the level of complexity needed in relation to the purpose. In the long term, the involvement of big data analysis and artificial intelligence (AI) may be relevant. A comprehensive database for cells, composite hydrogel components, and cell-material interactions need to be established from available knowledge. Based on the big data analysis, predictive models could be developed, while the design of composite hydrogels could be accelerated for the purpose of guiding cell fates in a relevant microenvironment of certain tissues and organs. Although cell behavior and tissue function tend to be the most important parameters to evaluate the suitability of composite hydrogels for 3D cultures, it is prudent for future studies to look at the remodeling and stability of the tissue matrix over time. The behavior and function of these cultures at steady state may be the true readout of success. Additionally, when non-endogenous elements such as metal and metal oxide nanomaterials are employed in composite hydrogels, it is prudent to understand the independent effects of these elements on cell and tissue physiology. When these are to be used for transplantation, the bio-persistence and potential implication on health will need to be addressed.

Looking ahead, validation of reliability and reproducibility of 3D tissue models will be critical for future adoption as standard testing platforms and for clinical transplantation. This is especially important for 3D culture models that rely on decellularized ECM or natural ECM components to guide cell fate, because these are often poorly defined with limited reproducibility due to batch-to-batch variations. Such variations could present significant reliability challenges when specific cell responses are anticipated (Chen and Liu, 2016). Using engineered biomaterials as hydrogel based cell matrices could improve reproducibility by having a more consistent raw material source. However, inconsistencies can be introduced during the fabrication process as well. For example, varying efficiencies of growth factor conjugation in different batches of hydrogels could result in significantly different levels of growth factor release and a consequent lack of reproducibility in cell response (Lee et al., 2011). Once an optimal composite hydrogel system has been identified, workflows to manufacture and screen for consistent quality on a large scale will need to be developed to encourage adoption. Finally, for the purposes of understanding system level interactions, it is timely to start thinking about developing interconnected 3D culture setups to study inter tissue/organ cross-talk (Maschmeyer et al., 2015; Skardal et al., 2016).

## Conclusion

Composite hydrogels have been developed to mimic the composition and structures of native tissues in 3D *in vitro* models. They are used as a tissue-like matrix to support cell functions, direct cell behaviors and tissue morphogenesis in the desired way for the development of tissue- and organ-like cultures. Composite hydrogels with reproducible properties have the potential to improve the efficiency and consistency of 3D cultures compared with traditional, single component matrices. Many material properties such as cell-binding capacity, matrix mechanics, and structural geometry would influence cell activities and are crucial design parameters for 3D cultures. As part of the additive manufacturing revolution, composite hydrogels have grown in popularity as bio-inks to print tissue-like models with encapsulated cells. Material properties that can impact their printability and resulting biological activities after 3D printing include viscosity, mechanisms of crosslinking, degradability, and presence of biological factors. Current proposed composite hydrogels are composed of combinations of natural and synthetic biomaterials, different types of nanomaterials, biological factors etc., which combine all the advantages of each constitute. In general, synthetic materials could provide chemical versatility and reproducible physicochemical properties, nanomaterials could improve mechanical properties, and natural biopolymers and biological macromolecules could provide cell readable ECM components. Thus, based on the properties of final composites desired, composite hydrogels provide a platform for designer matrices to be produced in the laboratory, in terms of types of constitutes, molecular weight of polymers, functional group engrafting, interaction of different materials (physical blending or chemical bonding), composition ratio and so on. This work reviewed recent important advances in composite hydrogels used for the creation of 3D *in vitro* models, and found that composite hydrogels are becoming the choice matrices for these. Looking ahead while there are still many technical challenges to be overcome, validating the reliability and reproducibility of specific composite hydrogels for specific 3D models will be crucial for adoption in therapeutic and pharmaceutical applications.

## Author Contributions

ZZ, CV-D, and KN conceptualized and planned the manuscript. ZZ, CV-D, ZM, MS, MR, and MK carried out the literature search and wrote specific sections. ZZ, CV-D, and KN edited the manuscript. All authors contributed to the article and approved the submitted version.

## Conflict of Interest

The authors declare that the research was conducted in the absence of any commercial or financial relationships that could be construed as a potential conflict of interest.
